# Visible-light induced degradation of diphenyl urea and polyethylene using polythiophene decorated CuFe_2_O_4_ nanohybrids

**DOI:** 10.1038/s41598-023-30669-x

**Published:** 2023-03-27

**Authors:** Ufana Riaz, Shayista Gaffar, Kristen Hauser, Fei Yan

**Affiliations:** 1 Department of Chemistry and Biochemistry, North Carolina Central University, Durham, NC 27707 USA; 2 Materials Research Laboratory, Department of Chemistry, Jamia Millia Islamia, New Delhi, 110025 India

**Keywords:** Chemistry, Materials science, Nanoscience and technology

## Abstract

The present work reports facile synthesis of CuFe_2_O_4_ nanoparticles via co-precipitation method and formulation of its nanohybrids with polythiophene (PTh). The structural and morphological properties were investigated using fourier transform infrared spectroscopy (FT-IR), X-ray diffraction (XRD), scanning electron microscopy coupled with energy dispersive spectra (SEM-EDS) and UV–Vis spectroscopy. The band gap was found to decrease with increase in the loading of PTh and was found to be 2.52 eV for 1-PTh/CuFe_2_O_4_, 2.15 eV for 3-PTh/CuFe_2_O_4_ and 1.89 eV for 5-PTh/CuFe_2_O_4_. The nanohybrids were utilized as photocatalysts for visible light induced degradation of diphenyl urea. Diphenyl urea showed 65% degradation using 150 mg catalyst within 120 min. Polyethylene (PE) was also degraded using these nanohybrids under visible light as well as microwave irradiation to compare its catalytic efficiency under both conditions. Almost 50% of PE was degraded under microwave and 22% under visible light irradiation using 5-PTh/CuFe_2_O_4_. The degraded diphenyl urea fragments were analyzed using LCMS and a tentative mechanism of degradation was proposed.

## Introduction

Urea is the most common nitrogen fertilizer used in agriculture worldwide and is generated in excess of 140 million tons annually^1^. About 650 tons of wastewater is discharged per day containing 0.5–2 wt% urea which is harmful to natural streams because it encourages the growth of algae and hydrolyzes slowly, releasing ammonia that is toxic to fish^2–4^. Besides this, many urea derivatives such as phenyl and sulfonyl urea are widely adopted as herbicides for the control of broad-leaved weeds and wild grasses in many agricultural crops. These compounds serve as persistent water pollutants due to their fairly high water solubility and relative photochemical stability. Likewise, plastic pollution has become a global problem and according to the US EPA (United States Environmental Protection Agency) more than 200 tons of plastic is produced annually^5^. Micro- plastics (MPs) (plastic materials with < 5 nm) that are directly discharged into the environment can build up in the stomach, induce blockage, inflammation in many organs and also produce genotoxicity^6–9^. The treatment of urea wastewater via biological^5,6^, enzymatic^10^ and electrochemical approaches have been attempted by several authors, while very few studies have been carried out on the degradation of plastics, including the bio/photocatalytic degradation of polyethylene polymers by *Zalerion maritimum*^11^ TiO_2_ nanotubes^12^, ZnO^13^, N- doped TiO_2_^14,15^, and Pt/ZnO^16,17^.

Lately, the photocatalytic properties of spinel ferrites have been widely reported for oxidative dehydration of hydrocarbons^18^, decomposition of alcohols and hydrogen peroxide^19^, oxidation of carbon monoxide^20^, and degradation of dyes^21–26^. Due to the alternation of Fe^+3^ and Fe^+2^ states, ferrites have been reported to exhibit remarkable structural stability as well as catalytic activity^22,23^. Conducting polymers such as polycarbazole (PCz)^22^, poly(o-phenylenediamine) (POPD)^24^, polypyrrole (PPy)^26^, polyaniline (PANI)^27^, polythiophene (PTh)^28^, are widely known to act as sensitizers and reduce the band gap of the metal oxides. Lately few authors have reported the degradation of polymers using spinels^29,30^. However, no work has been reported on the utilization of CuFe_2_O_4_ in the degradation of diphenyl urea/plastics. Hence, with a view to explore the photocatalytic efficiency of conducting polymer-sensitized nanohybrids in the degradation of plastics as well as diphenyl urea which are seldom investigated persistent organic pollutants, we have synthesized magnetic CuFe_2_O_4_ nanoparticles and formulated its nanohybrids using different loadings of PTh. The synthesized nanomaterials were characterized for their spectral, thermal, and morphological properties. CuFe_2_O_4_ and PTh/CuFe_2_O_4_ nanohybrids were chosen to evaluate the degradation efficiency of diphenyl urea and PE. The degradation of diphenyl urea was systematically investigated by varying the effect of urea as well as the nanohybrid concentration. The reusability of CuFe_2_O_4_ and CuFe_2_O_4_/PTh nanohybrids were evaluated and the mechanism of degradation was proposed based on the radical scavenging experiments and LCMS studies. The degradation of PE was studied under visible light irradiation as well as microwave irradiation and the degradation was confirmed via weight loss studies and IR analysis.

## Materials and methods

### Materials

Cupric chloride (CuCl_2_), (Merck, India), ferric chloride (FeCl_3_) (Sigma Aldrich, USA), Ammonia (Sigma Aldrich, USA), thiophene (Sigma Aldrich, USA), tert-butanol (TBA) (Merck, India), disodium sulfate (Na_2_SO_4_) (Sigma Aldrich, USA), ethylene diamine tetra acetate (EDTA) (Sigma Aldrich, USA), sodium nitrate (Sigma Aldrich, USA), ethanol (Merck, India), chloroform (Merck, India), were used without further purification. Urea (CH_4_N_2_O) (molar mass 60.06 g/mol) (Merck, India) was used as received.

### Synthesis of CuFe_2_O_4_ nanoparticles

Cupric chloride (CuCl_2_) (2 g) and FeCl_3_ (4 g) were dissolved in distilled water (50 ml) under vigorous magnetic stirring at room temperature (25 °C) for 30 min. A solution of ammonia (3 M, 25 ml) was added drop-wise into the above mixture under constant stirring on a magnetic stirrer for 1 h to attain a pH level of 11–12. The solution was further heated at 80 °C for 2 h and the product was cooled to room temperature after 1 h. The precipitate was collected via centrifugation for 5 min at 300 rpm, washed with ethanol to remove unreacted precursors and then dried in vacuum oven at 120 °C for 12 h. After drying, the precipitate was subjected to calcination at 500 °C for 3 h in a muffle furnace to obtain CuFe_2_O_4_ nanoparticles.

### Synthesis of PTh modified CuFe_2_O_4_ nanohybrids

CuFe_2_O_4_ nanoparticles (1 g) were dispersed in a solution of CHCl_3_:H_2_O mixture (30 ml/30 ml v/v) in a 150 ml conical flask and thiophene monomer (0.01 ml) was added to the same flask. The reaction mixture was sonicated at 25 °C on an ultrasonic bath (30 kHz) for 30 min. Ferric chloride (0.019 g) (thiophene is to FeCl_3_ ratio 1:1) dissolved in CHCl_3_ (30 ml) was then added drop wise to the above reaction mixture and subjected to further sonication for 4 h. The obtained nanohybrid was filtered, washed several times with distilled water and dried in vacuum oven for 24 h at 80 °C. Based on the weight ratios of the ferrite/monomer taken, the nanohybrids were designated as 1-PTh/CuFe_2_O_4_, 3-PTh/CuFe_2_O_4_ and 5-PTh/CuFe_2_O_4_.

### Characterization

#### Morphological analysis

The XRD profiles were recorded on Philips PW 3710 powder X-ray diffractometer using Ni-filtered Cu-Kα radiation. The morphology and microstructures of the synthesized samples were investigated by field emission-scanning electron microscopy (FE-SEM) (FEI, Nova nano SEM 30 KVA, Germany).

#### Spectral analysis

The IR spectra were recorded on the FTIR spectrophotometer (Perkin Elmer, USA) in the form of powder. The UV–Vis diffuse reflectance (UV-DRS) spectra were captured on a PerkinElmer Lambda 30 spectrophotometer using BaSO_4_ as a reference.

#### Visible light induced degradation studies

Degradation experiments were carried out using xenon lamp light source (500 W, CEL-S500F, Beijing, China) (spectrum provided in supporting information as Fig. S1). Approximately 150 mg of the catalyst was dispersed in diphenyl urea solution (300 ml) and was sonicated for 10 min. The suspension was then kept under dark conditions to stabilize the adsorption–desorption equilibrium between the diphenyl urea solution and catalyst. The diphenyl urea solution was exposed to visible irradiation for 120 min and aliquots of solution (3 ml) were taken out at regular intervals of 0, 20, 40, 60, 80, 100 and 120 min. The UV spectrum of the diphenyl urea was recorded using UV–Vis spectrophotometer model Perkin-Elmer Lambda 30 at λ_max_ value of 250 nm.

#### Scavenging experiments for analysis of radical responsible for diphenyl urea degradation

The reactive species responsible for the degradation of diphenyl urea were investigated as per method reported in our previous studies using 5 mM of scavengers namely tert-butanol (TBA) (for ^·^OH radical detection), sodium nitrate (for O_2_^·−^ radical detection), ethylene diamine tetra acetate (EDTA) (for hole (h^+^) detection) and disodium sulfate (Na_2_SO_4_) (for electron (e^−^) detection)^31,32^. To study the impact of the scavengers on the degradation rate, the scavengers were added one at a time to the urea solution containing the catalyst (50 mg).

#### Recyclability tests for urea degradation

The reusability of CuFe_2_O_4_ and PTh/CuFe_2_O_4_ nanohybrids were evaluated for the degradation process up to four cycles. The nanohybrids CuFe_2_O_4_ and CuFe_2_O_4_/PTh nanohybrids were collected after completion of each cycle, washed with distilled water and dried under vacuum oven at 60 °C for 6 h. The obtained catalysts were reused in the next cycle and the process was repeated three times.

## Results and discussion

### Structural confirmation of CuFe_2_O_4_ and CuFe_2_O_4_/PTh nanohybrids via XRD and SEM studies

**Figure 1:**
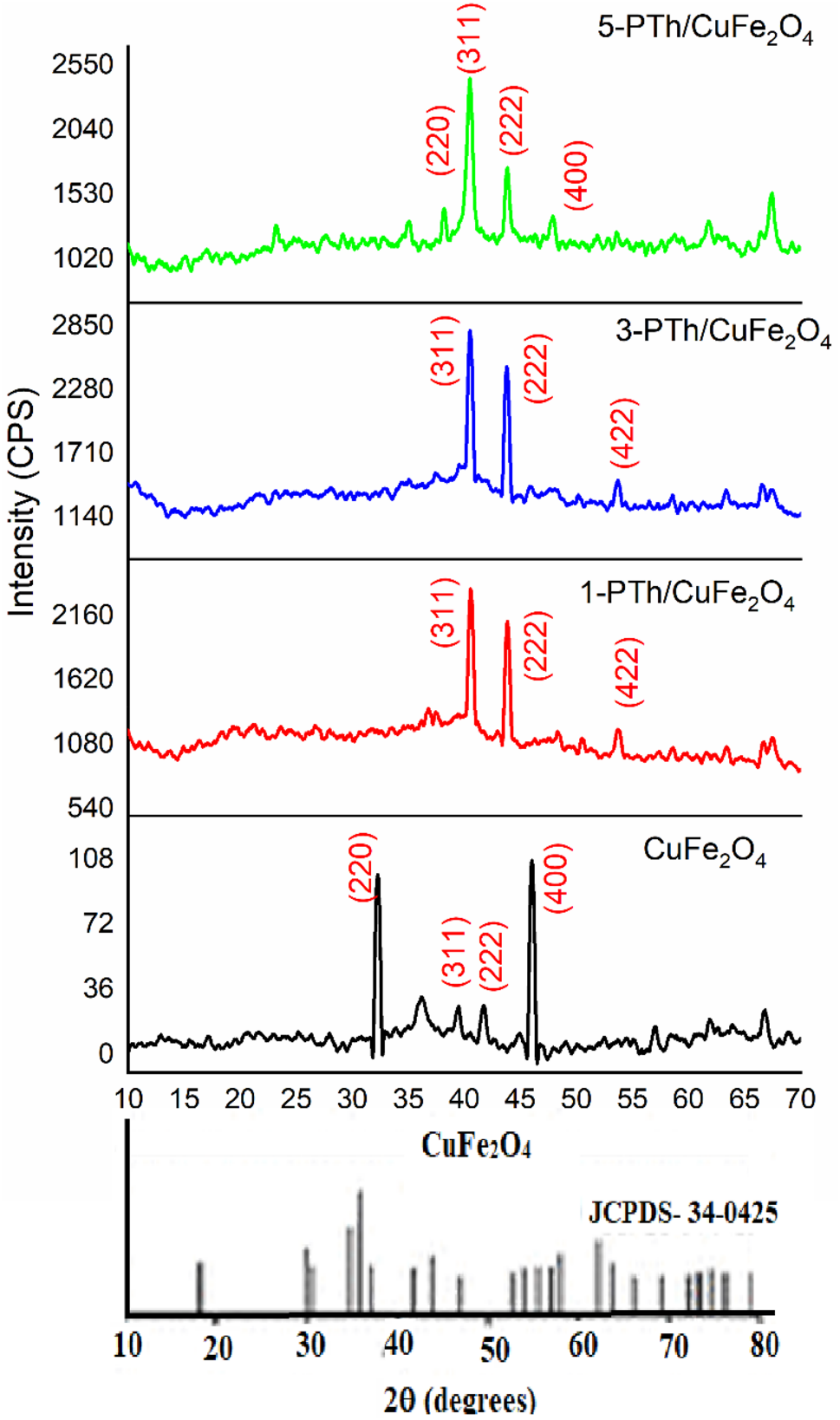
XRD profile of CuFe_2_O_4_, 1-PTh/CuFe_2_O_4_, 3-PTh/CuFe_2_O_4_, and 5-PTh/CuFe_2_O_4_.

The XRD profile of CuFe_2_O_4_, Fig. 1, revealed diffraction peaks at 2θ = 32.14°, 39.42°, 41.82°, 46.04° with Miller indices (220), (311), (222), and (400) respectively, which matched very well with the characteristic peaks of CuFe_2_O_4_ and with the space group of Fd3m^33^. The peaks matched well with the cubic phase of CuFe_2_O_4_ as per JCPDS card no 34-0425. The reflection peaks with miller indices (220) and (400) were found to be most intense and were associated with the characteristic peaks of spinel phase. The reflection peak patterns were coherent with single phase cubic structure confirming the solubility of Cu^2+^ into Fe_3_O_4_ and formation of well-crystallized nanostructure^33,34^. The average grain size was estimated to be 45 ˚A using the Debye–Scherrer formula^35^. The nanohybrids revealed prominent reflection peaks corresponding to CuFe_2_O_4_ at 40.6° and 43.8° with Miller indices (311) and (222). The peaks were found to be more prominent than the ones with Miller indices (220) and (400) presumably due to the change in the orientation of the planes upon loading of PTh. Since no other peaks associated with the presence of PTh were noticed, the nanohybrids were confirmed to be encapsulated with the polymer. The crystallinity of CuFe_2_O_4_ spinel was noticed to remain intact even upon loading of the polymer up to 5% in the nanohybrids. The encapsulation of the spinel by the polymer was further confirmed by the morphological studies discussed in the proceeding section.

### SEM analysis

**Figure 2:**
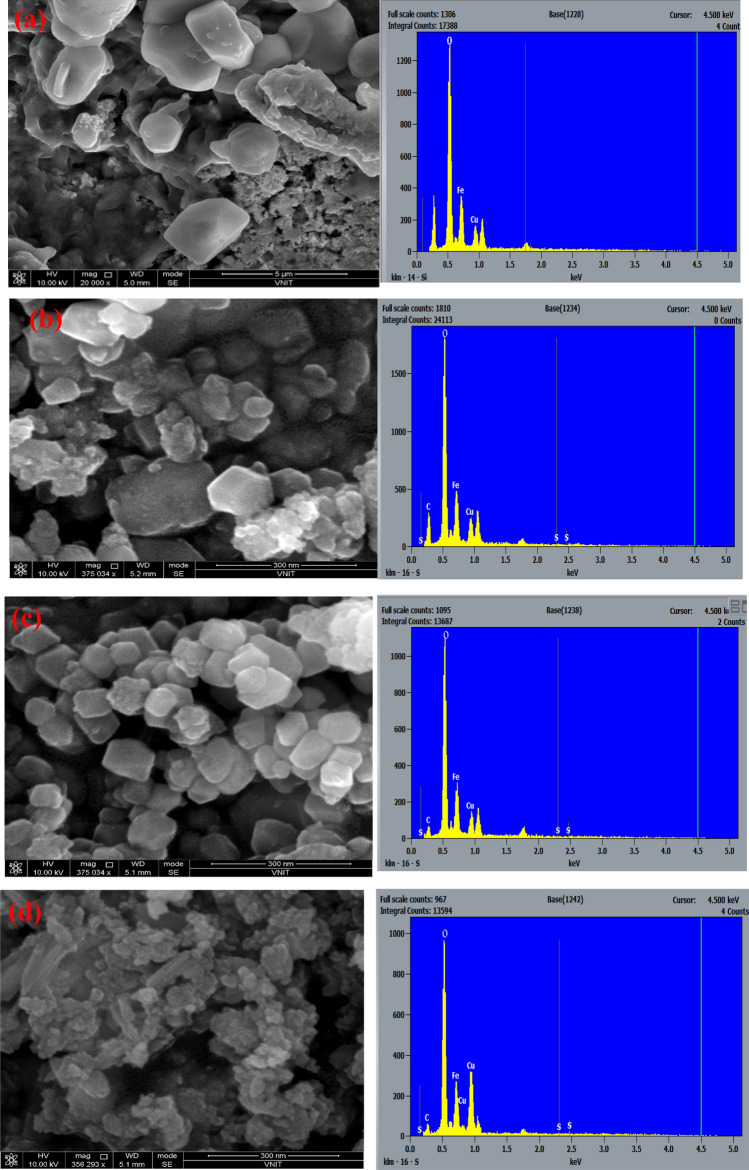
SEM image of (**a**) CuFe_2_O_4_ (**b**) 1-Th/CuFe_2_O_4_ (**c**) 3-PTh/CuFe_2_O_4_ (**d**) 5-PTh/CuFe_2_O_4_.

The morphological studies of as prepared CuFe_2_O_4_ nanoparticles and CuFe_2_O_4_/PTh nanohybrids were investigated by SEM, Fig. 2a–d. The SEM image of CuFe_2_O_4_, Fig. 2a, exhibited the formation of bright distorted cubes. The SEM of 1-PTh/CuFe_2_O_4_ nanohybrid, Fig. 2b, showed granular morphology, while the SEM of 3-PTh/CuFe_2_O_4_, Fig. 2c, revealed the presence of cubical clusters of CuFe_2_O_4_ and granular particles which were associated with PTh. The SEM of 5-PTh/CuFe_2_O_4_, Fig. 2d, showed presence of distorted bright cubes enclosed with PTh dark granular aggregates. There was slight variation noticed in the particle sizes upon increasing the loading of PTh in CuFe_2_O_4_. Thus, it can be inferred that the nanohybrids revealed encapsulation of CuFe_2_O_4_ with spherical PTh particles forming dense dark agglomerates. The elemental composition of CuFe_2_O_4_ and CuFe_2_O_4_/PTh nanohybrids was determined by EDS analysis and showed the presence of Cu, Fe and O with stoichiometric values matching that of CuFe_2_O_4_ composition (Fig. 2a), whereas the EDS spectrum of CuFe_2_O_4_/PTh nanohybrids showed the presence of C and S in addition to Cu, Fe and O, Fig. 2b–d.

### IR studies

The IR spectra of CuFe_2_O_4_ and its nanohybrids CuFe_2_O_4_/PTh were also studied (given in supporting information as Fig. S2). The IR spectrum of CuFe_2_O_4_ showed OH stretching vibration peak at 3123 cm^−1^ correlated to the presence of adsorbed water molecules. The peak at 1215 cm^−1^ was correlated Fe–Cu bond stretching vibration mode, while the peaks related to the Cu–OH bond vibration were noticed at 1069 cm^−1^. The peak related to Fe–O stretching was noticed at 789 cm^−1^, whereas peaks at 550 cm^−1^, and 486 cm^−1^ were correlated to the stretching vibrations of Cu–O. The presence of the above peaks confirmed the formation of CuFe_2_O_4_^23,36^._._ The IR spectrum of PTh/CuFe_2_O_4_ nanohybrids showed a peak at 3121 cm^−1^ attributed to the O–H stretching vibration, while the peak at 1739 cm^−1^ was associated with the O–H bending vibrations of absorbed water molecules. The peaks at 1607 cm^−1^ and 1511 cm^−1^ was correlated to the C=C stretching vibrations in the thiophene ring molecules. The peak at 1215 cm^−1^ was assigned to the C–H bending vibrations. The characteristic peaks at 1117 cm^−1^ and 879 cm^−1^ in IR spectrum of nanohybrids were assigned to the C–S stretching vibrations. The absorption band at 788 cm^−1^ was associated with C–H out of plane stretching vibrations and C–H plane deformation modes of PTh^28^. The peaks at 571 cm^−1^ was also observed due to ring deformation of PTh. The presence of the peaks related to spinel as well as the polymer confirmed the formation of CuFe_2_O_4_ and the presence of PTh in CuFe_2_O_4_/PTh nanohybrids.

### UV analysis and confirmation of band gap

Ferrites exhibit photocatalytic activity by the absorption of visible light, which leads to the transfer of electrons from the valence band to the conduction band, creating electrons and holes. CuFe_2_O_4_ showed an absorption edge of around 450 nm, while the nanohybrids revealed shift in the absorption edge to 550 nm and higher with increase in the PTh loading to 5% (given in supporting information as Fig. S3). Hence, it can be concluded that the nanohybrids can efficiently absorb the light in the visible region. The Kubelka–Munk equation was applied to calculate the optical band gap^31,32,36^. The band gap values were found to be 2.74 eV for CuFe_2_O_4_, 2.52 eV for 1-PTh/CuFe_2_O_4_, 2.15 eV for 3-PTh/CuFe_2_O_4_, 1.89 eV for 5-PTh/CuFe_2_O_4_ (given in supporting information as Fig. S4a–d). The band gap of CuFe_2_O_4_ showed a consistent decrease with an increase in the amount of PTh in the CuFe_2_O_4_ and was suitable for degrading diphenyl urea under visible light as discussed in the proceeding section.

### Effect of urea concentration and photocatalyst concentration on degradation

**Figure 3:**
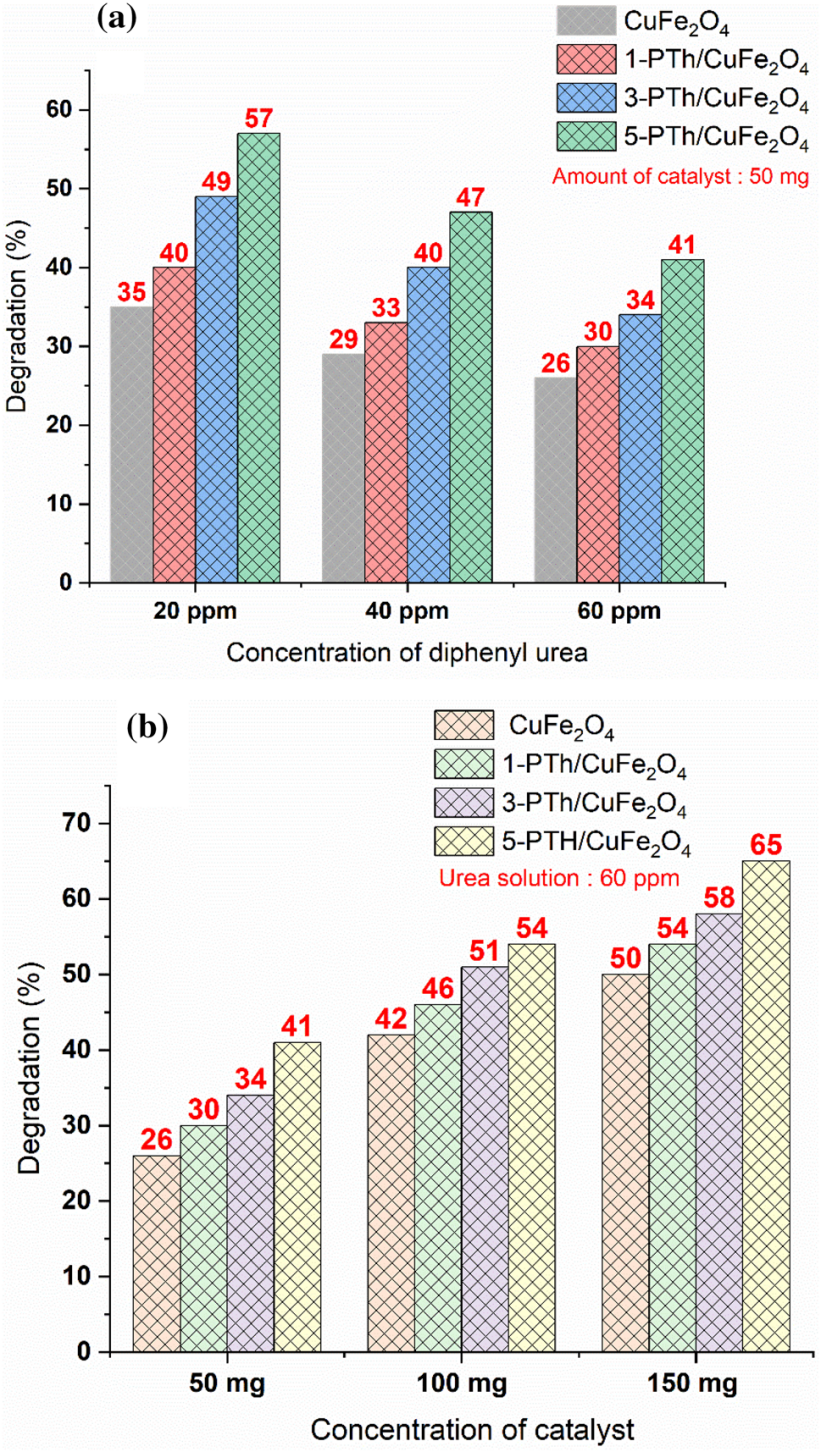
(**a**) Effect of diphenyl urea concentration (**b**) Effect of photocatalyst concentration on the degradation behavior of the pollutant.

Degradation of diphenyl urea was performed by visible light irradiation for a period of 120 min and was examined by UV–Vis spectroscopy. To study the effect of urea concentration, 20 ppm, 40 ppm and 60 ppm solutions were taken along with 50 mg of the photocatalyst and exposed to visible light irradiation for a period of 120 min as depicted in Fig. 3a. Almost 35% degradation of 20 ppm diphenyl urea solution was achieved in 120 min using 50 mg CuFe_2_O_4_, and 29% degradation was attained for 40 ppm solution using the same amount of the photocatalyst. The 60 ppm solution showed 26% degradation efficiency within 120 min. The rate constant (k) values for 20 ppm, 40 ppm and 60 ppm degradation were calculated to be 0.0036 min^−1^, 0.0029 min^−1^ and 0.0026 min^−1^ respectively (given in supporting information as Fig. S5a–d). The nanohybrid 1-PTh/CuFe_2_O_4_ showed 30% degradation of 60 ppm solution within a span of 120 min, while almost 40% and 33% degradation was noticed for 20 ppm and 40 ppm diphenyl urea solutions respectively, Fig. 3a. The k values for 20 ppm, 40 ppm and 60 ppm degradation were found to be 0.0044 min^−1^, 0.0033 min^−1^ and 0.0031 min^−1^ respectively (given in supporting information as Fig. S5a–d). The nanohybrids 3-PTh/CuFe_2_O_4_ exhibited 49% degradation for 20 ppm diphenyl urea solution; while around 40% degradation of 40 ppm diphenyl urea solution was achieved in 120 min and 34% for 60 ppm solution. The rate constant values for 20 ppm, 40 ppm and 60 ppm were found to be 0.0058 min^−1^, 0.0043 min^−1^ and 0.0037 min^−1^ respectively. The nanohybrids 5-PTh/CuFe_2_O_4_ revealed of 57% , 47% and 41% degradation of 20 ppm, 40 ppm and 60 ppm urea solutions and the k values were observed to be 0.0072 min^−1^, 0.0055 min^−1^ and 0.0046 min^−1^ respectively. Among the nanohybrids, 5-PTh/CuFe_2_O_4_ showed highest degradation efficiency of 57% within 120 min for 20 ppm diphenyl urea solution.

To study the effect of catalyst concentration, CuFe_2_O_4_ and PTh/CuFe_2_O_4_ nanohybrids were taken as 50 mg, 100 mg and 150 mg for degradation of 60 ppm urea solution and are depicted in Fig. 3b. With the increase in catalyst concentration, the degradation efficiency was found to increase rapidly. For CuFe_2_O_4_, the degradation was observed to be 26%, 42% and 50% using 50 mg, 100 mg and 150 mg of the photocatalyst respectively. The k values were noticed to be 0.0026 min^−1^, 0.0046 min^−1^ and 0.0055 min^−1^ respectively, (given in supporting information as Fig. S6a–d). Similarly, 1-PTh/CuFe_2_O_4_, exhibited 30%, 46% and 54% degradation with the increase in the concentration of the photocatalyst. The k values were found to be 0.0031 min^−1^, 0.0052 min^−1^, and 0.0064 min^−1^. The nanohybrid 3-PTh/CuFe_2_O_4_ showed 34% degradation in presence of 50 mg photocatalyst, while almost 58% degradation was attained using 150 mg photocatalyst and 51% using 100 mg catalyst. The k values were noticed to be 0.0037 min^−1^, 0.0059 min^−1^ and 0.0074 min^−1^ respectively for 50 mg, 100 mg and 150 mg photo catalysts (given in supporting information as Fig. S6a–d). Likewise, the nanohybrid 5-PTh/CuFe_2_O_4_ showed 41% degradation in presence of 50 mg photocatalyst, while almost 54% degradation was attained using 100 mg photocatalyst and 65% using 150 mg catalyst. The k values were noticed to be 0.0046 min^−1^, 0.0065 min^−1^ and 0.0088 min^−1^ respectively for 50 mg,100 mg and 150 mg photocatalysts.

### Radical scavenging experiments

**Figure 4:**
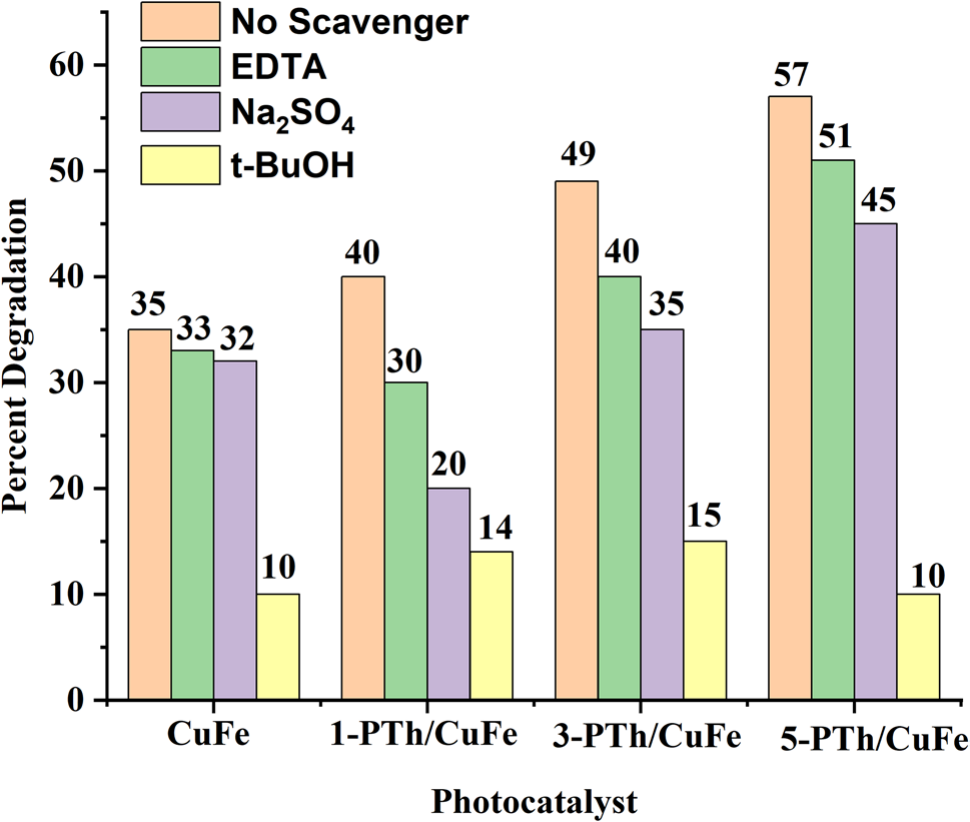
Effect of addition of radical scavenger on CuFe_2_O_4_, PTh/CuFe_2_O_4_ nanohybrids.

Radical trapping experiments were carried out to identify the primary reactive oxidative species involved in the photo degradation of the diphenyl urea in order to investigate the mechanism of photocatalysis in presence of CuFe_2_O_4_ and CuFe_2_O_4_/PTh nanohybrids. Different scavengers sodium sulfate (Na_2_SO_4_) (e^−^ scavenger) (5 ml, 5 Mm), ethylene diamine tetra acetic acid (EDTA) (h^+^ scavenger) (5 ml 5 Mm), and t-butyl alcohol (t-BuOH) (^**·**^OH scavenger) (5 ml, 5 Mm) were added into urea solution and exposed to visible irradiation for 120 min in presence of CuFe_2_O_4_ and its nanohybrids, Fig. 4. The spinel CuFe_2_O_4_, in presence of EDTA, showed a decrease in the degradation efficiency of urea to 33%, while in presence of Na_2_SO_4_, the degradation efficiency decreased to 32%. However, in presence of t-BuOH, the degradation efficiency was noticed to be 10% confirming the generation of ^·^OH radicals as the major active species. For 1-PTh/CuFe_2_O_4_, the degradation efficiency was observed to be 30% in presence of EDTA and 20%, 14% for Na_2_SO_4_ and t-BuOH respectively.

**Figure 5:**
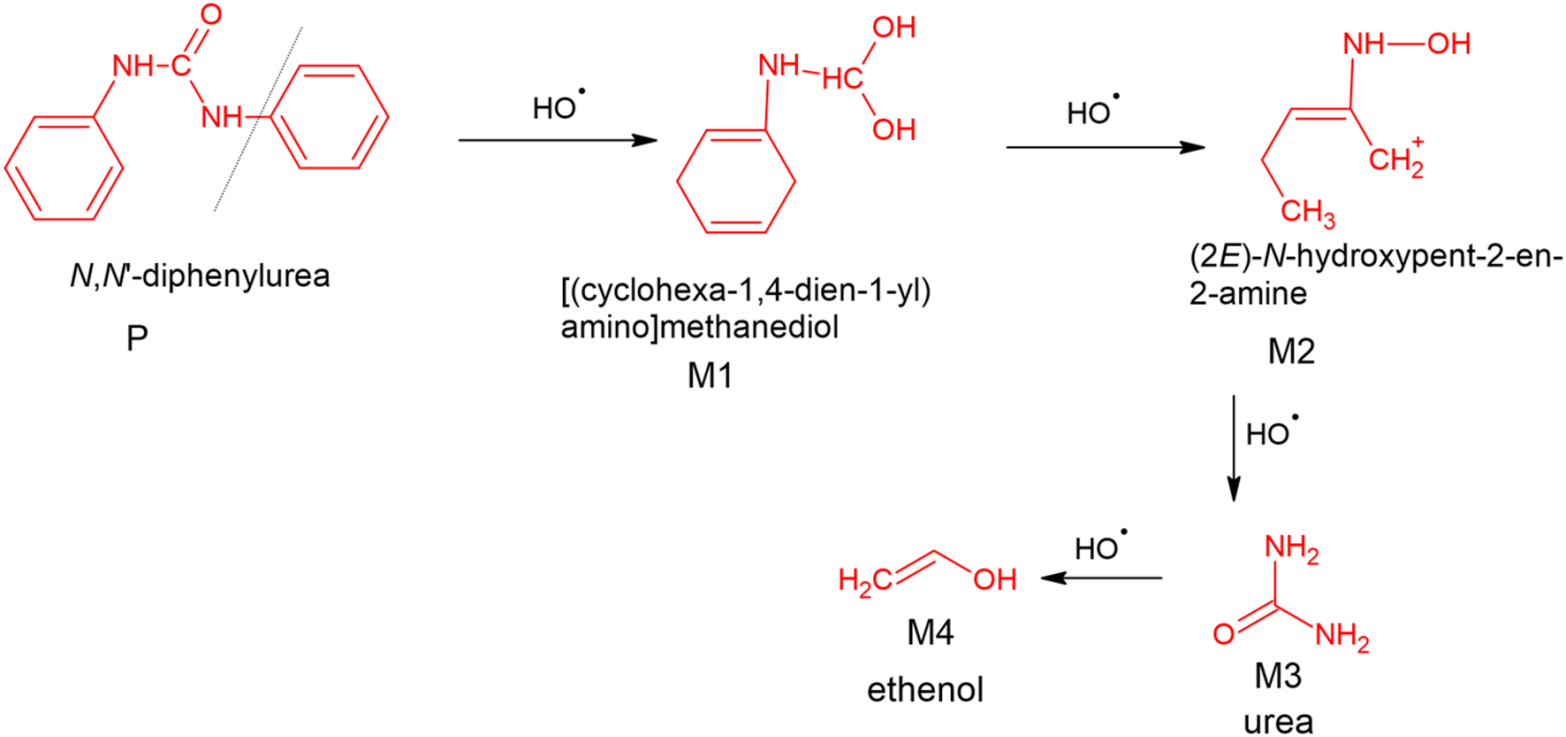
Mechanism of degradation of Diphenyl urea.

Similarly, for 3-PTh/CuFe_2_O_4_, the degradation efficiency was reduced to 40% and 35% upon addition of EDTA and Na_2_SO_4_ respectively and 15% upon addition of t-BuOH. The nanohybrid, 5-PTh/CuFe_2_O_4_, revealed a degradation efficiency of  51% and 45% in presence of EDTA and Na_2_SO_4_ respectively and 10% in presence of t-BuOH. The reduction in the degradation rate in presence of t-BuOH in all cases confirmed that ^·^OH radicals were the main active radical species as compared to e^−^ and h^+^ involved in the photocatalytic degradation of diphenyl urea using CuFe_2_O_4_ and PTh/CuFe_2_O_4_ as photocatalysts. The visible light induced degradation of diphenyl urea using CuFe_2_O_4_ and PTh/CuFe_2_O_4_ nanohybrids generate radicals which lead to the excitation of electrons from the valence band to the conduction band and PTh acts as a sensitizer to promote electrons in the conduction band of CuFe_2_O_4_ creating holes (h^+^) in the VB of the later. As confirmed from the radical scavenging studies, the generation of hydroxyl radicals (·OH), causes degradation of diphenyl urea. The LCMS study (given in supporting information as Fig. S7) of the degraded fragments of diphenyl urea was used to propose the tentative degradation pathway of diphenyl urea as shown in Fig. 5. The diphenyl urea parent molecule upon exposure to hydroxyl radical fragmented to [(cyclohexa-1,4-dien-1-yl) amino] methanediol (M1) (m/z 141) which further fragmented to (2*E*)-*N*-hydroxypent-2-en-2-amine (M1) (m/z 100), urea (M3) (m/z 60) and ethenol (M4) (m/z 44).

### Recyclability test

**Figure 6:**
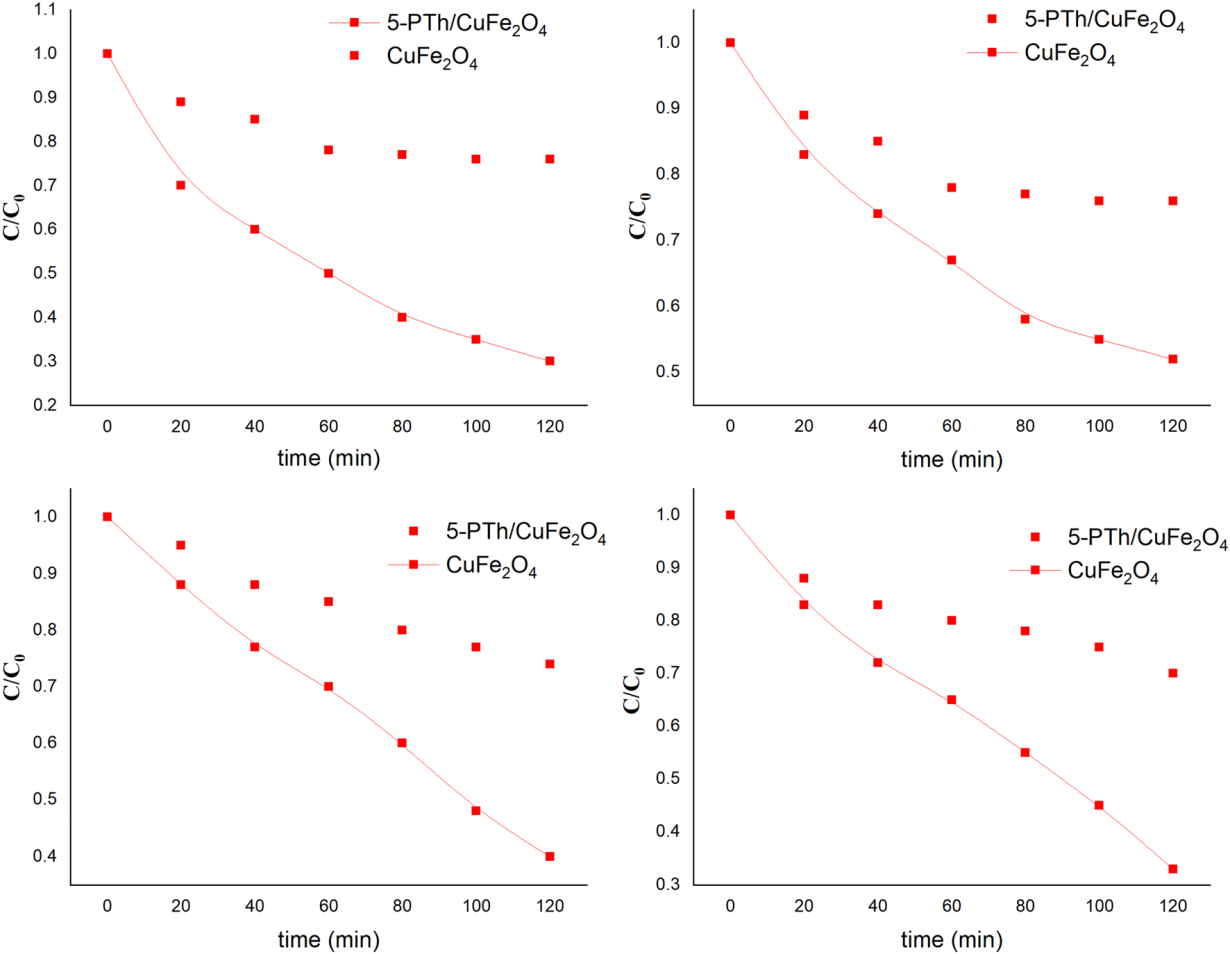
Recyclability test using CuFe_2_O_4_ and 5-PTh/CuFe_2_O_4_.

The recyclability tests, Fig. 6 showed that CuFe_2_O_4_ and 5% PTh-CuFe_2_O_4_ taken as 50 mg revealed 35% and 60% degradation of 20 ppm urea solution exhibiting high catalytic activity even after four cycles which confirmed their excellent reusability as well as stability. Therefore, 5% PTh-CuFe_2_O_4_ could be considered as a potential catalyst in wastewater treatment due to its fairly high reusability feature.

### Comparison of visible light and microwave-assisted degradation of polyethylene

**Figure 7:**
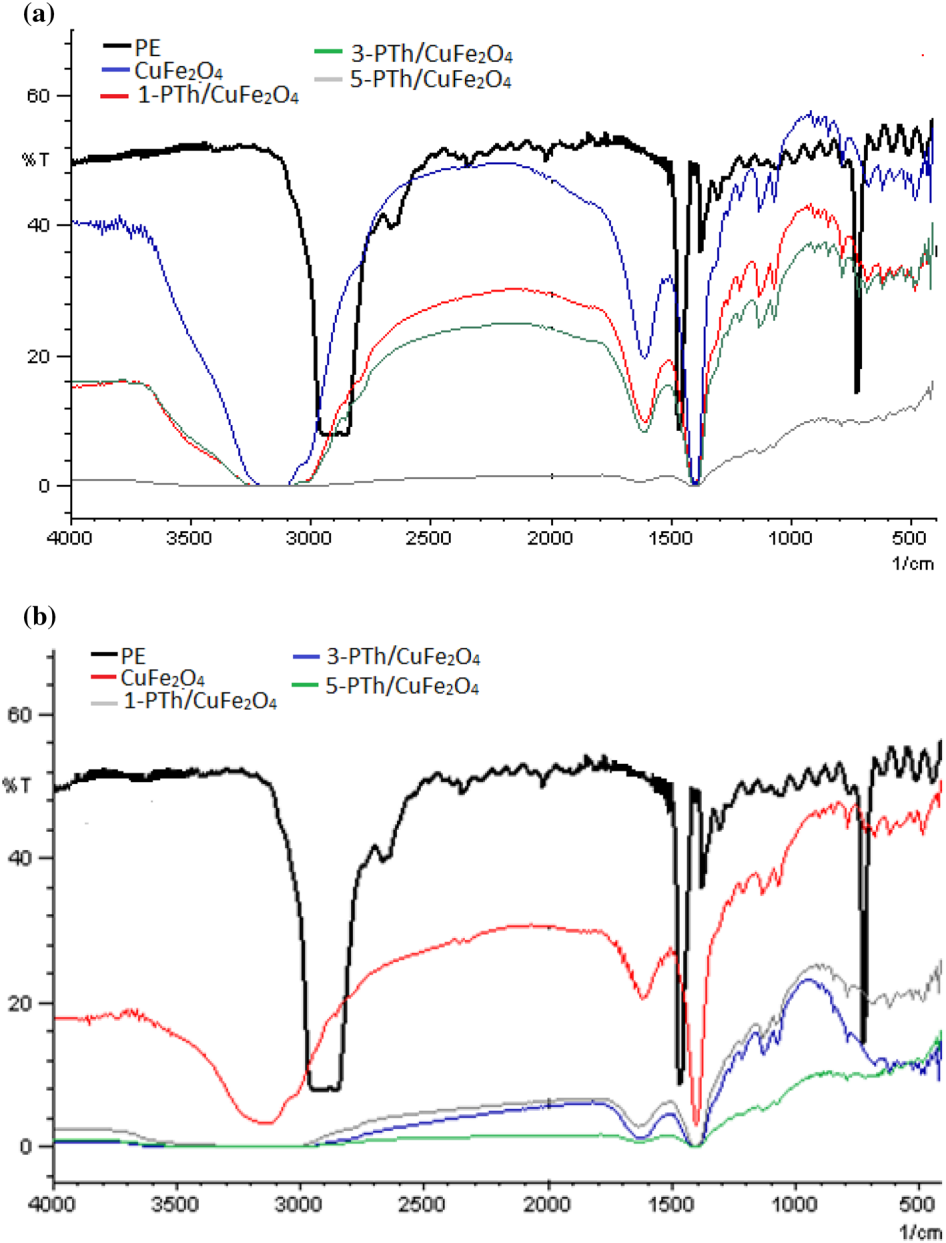
FTIR of (**a**) photo-catalytically degraded PE films (**b**) microwave degraded PE films.

Polyethylene films (PE) were cut into strips (1 cm × 1 cm) and were subjected to visible light induced degradation using a solution of (water/DMSO) solvent. Similarly, for microwave induced degradation, the solution containing the films was degraded under microwave irradiation (Ladd Research Microwave oven) using 150 mg of CuFe_2_O_4_ as well as PTh/CuFe_2_O_4_ as catalyst and the results are shown in Fig. 7a and b.

The IR studies reveal that a major shift as well as disappearance of the peaks was observed upon photocatalytic degradation of the PE films in presence of PTh/CuFe_2_O_4_ as catalyst. The IR peaks of PE film before degradation were noticed at 2914 cm^−1^, 2845 cm^−1^, 1465 cm^−1^, 1370 cm^−1^, 719 cm^−1^^37^. Upon exposure to microwave irradiation with photocatalyst for 5 h, the peaks were found to show a major shift. A shift in peaks at 1466 cm^−1^, 1370 cm^−1^ was observed towards 1600 cm^−1^. The initial weight of PE was found to be 0.004 g, but after degradation decreased appreciably as shown in Table 1. The degradation was observed to be 10% using CuFe_2_O_4_ in presence of visible light and 28% under microwave irradiation. The degradation of PE films was found to be highest using 5-PTh/CuFe_2_O_4_ which was noticed to be almost 50% under microwave irradiation. It can thus be concluded that the nanohybrids could be effectively used to degrade polymers under microwave irradiation.

**Table 1:** Percentage of weight loss in degraded PE via photocatalysis and microwave degradation.

Catalyst used	Initial weight (g)	Final weight (g) PE by photocatalysis	Final weight (g) PE microwave	% wt. loss PE by photocatalysis	% wt. loss PE by microwave
CuFe_2_O_4_	0.004 g	0.0036 g	0.0029 g	10%	27.5%
1-PTh/CuFe_2_O_4_	0.004 g	0.0034 g	0.0027 g	15%	32.5%
3-PTh/CuFe_2_O_4_	0.004 g	0.0032 g	0.0024 g	20%	40%
5-PTh/CuFe_2_O_4_	0.004 g	0.0031 g	0.0020 g	22.5%	50%

## Conclusion

CuFe_2_O_4_ and PTh/CuFe_2_O_4_ nanohybrids were successfully prepared and characterized for their spectral as well as morphological properties. The XRD analysis confirmed the formation of CuFe_2_O_4_ spinel with cubic phase which undergoes encapsulation upon loading of PTh. The UV-DRS studies showed appreciable reduction in band gap values upon modification with PTh which confirmed that the nanohybrids could effectively be used for visible-light-induced photocatalysis. The visible-light induced photocatalytic degradation of diphenyl urea was carried out for 120 min, while microwave-assisted as well as visible light induced catalytic degradation of PE films was carried out for 5 h. For the visible light-induced degradation of diphenyl urea, the kinetics fitted the first-order model and was found to be 57% for the 20 ppm solution using 5-PTh/CuFe_2_O_4_ nanohybrid. The recyclability tests showed that the photocatalyst could be safely used up to a maximum of four cycles. For the PE films, a maximum degradation of 50% was achieved under microwave irradiation for 5 h using 5-PTh/CuFe_2_O_4_ nanohybrid. The photocatalyst could be effectively used for visible light-induced degradation of pollutants as well as microwave-assisted degradation of polymers.

## Supplementary Information

Supplementary Figures.

## Data Availability

All data generated or analysed during this study are included in this published article [and its supplementary information files.
