# Supplementary material for: Visible-light induced degradation of diphenyl urea and polyethylene using polythiophene decorated CuFe2O4 nanohybrids
Source: Sci Rep. 2023 Mar 27;13:4975. doi: 10.1038/s41598-023-30669-x (PMC10043026; doi:10.1038/s41598-023-30669-x)
Supplement: Supplementary file 1 — Supplementary Figures. [file 41598_2023_30669_MOESM1_ESM.docx]

s

sss

**Supporting Information**

**Visible-Light Induced Degradation of Diphenyl Urea and Polyethylene using Polythiophene Decorated CuFe_2_O_4_ Nanohybrids**

**Ufana Riaz^a,b^∗ ,Shayista Gaffar^b^ , Kristen Hauser^a^, Fei Yan^a^**

**^a^Department of Chemistry and Biochemistry, North Carolina Central University, NC, 27707, USA**

**^b^Materials Research Laboratory, Department of Chemistry, Jamia Millia Islamia,**

**New Delhi 110025, India, *Corresponding author: Fax-(+91-112-684-0229); E-mail address- (**[**ufana2002@yahoo.co.in**](mailto:ufana2002@yahoo.co.in)**,uriaz@nccu.edu)**


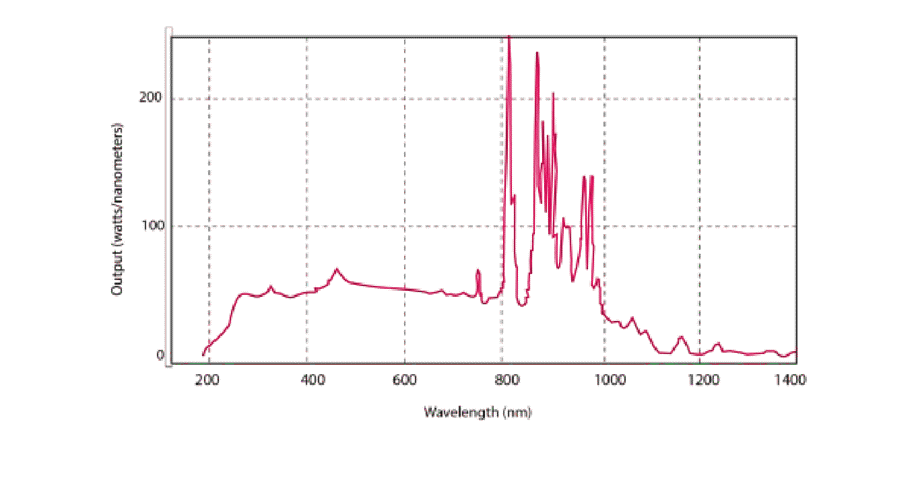


Figure

Figure

**Figure S1 Power spectrum of Xenon lamp used for degradation studies**

**
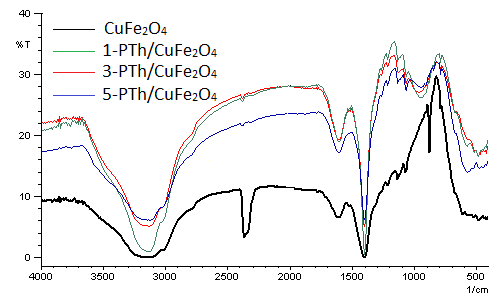
**

**Figure S2 IR spectra of CuFe_2_O_4_ and PTh/CuFe_2_O_4_ nanohybrids**

**

**

**Figure S3 Reflectance spectra of CuFe_2_O_4_, 1-PTh/CuFe_2_O_4_, 3-PTh/CuFe_2_O_4_ and 5- PTh/CuFe_2_O_4_**













**Figure S4 Band gap values of (a) CuFe_2_O_4_ (b) 1-PTh/CuFe_2_O_4_  (c) 3-PTh/CuFe_2_O_4_ (d) 5-PTh/CuFe_2_O_4_ nanohybrids**

**
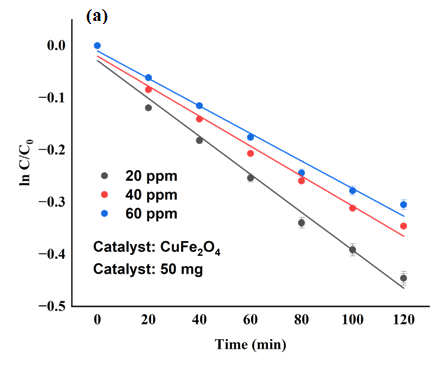
**

**
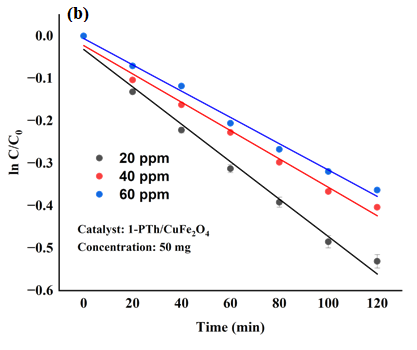
**

**
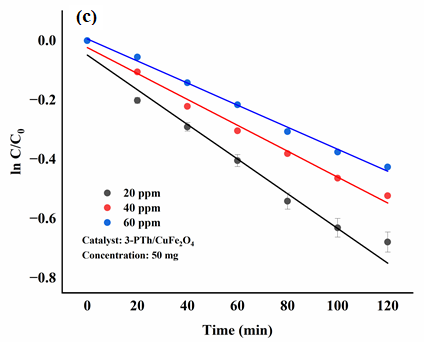
**

**
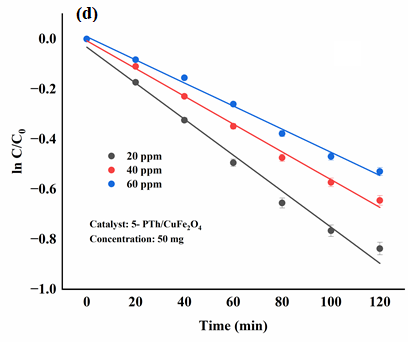
**

**Figure S5 Effect of urea concentration, ln C/C_o_ plots for (a) CuFe_2_O_4_, (b) 1-PTh/CuFe_2_O_4_, (c) 3- PTh/CuFe_2_O_4_, (d) 5- PTh/CuFe_2_O_4_ as photocatalysts**


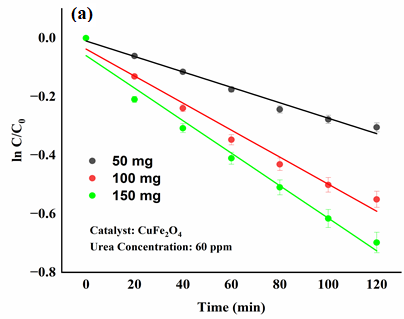


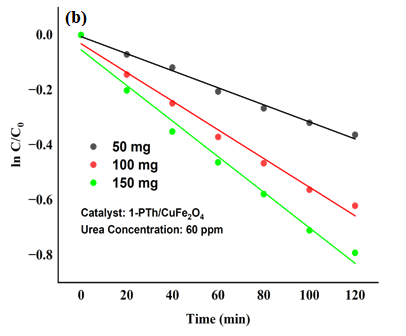


**
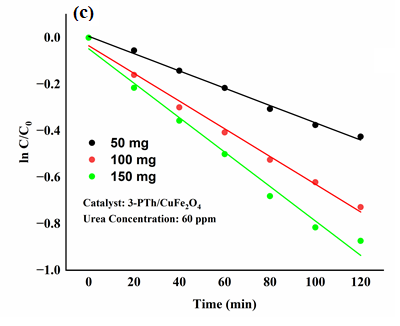
**

**
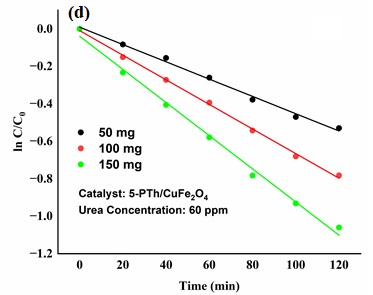
**

**Figure S6 Effect of photocatalyst concentration, on the degradation of diphenyl urea using (a) CuFe_2_O_4_, (b) 1-PTh/CuFe_2_O_4_, (c) 3- PTh/CuFe_2_O_4_, (d) 5- PTh/CuFe_2_O_4_ nanohybrids**

**
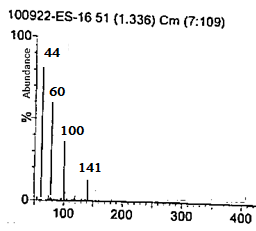
**

**Figure S7 LCMS data of degraded diphenyl urea**
